# Editorial: Age-Related Changes in Body Composition: Mechanisms, Clinical Implications and Possible Treatments

**DOI:** 10.3389/fmed.2020.00230

**Published:** 2020-07-14

**Authors:** Anna Picca, Francesco Landi, Matteo Cesari

**Affiliations:** ^1^Fondazione Policlinico Universitario “Agostino Gemelli” IRCCS, Rome, Italy; ^2^Institute of Internal Medicine and Geriatrics, Università Cattolica del Sacro Cuore, Rome, Italy; ^3^Department of Clinical Sciences and Community Health, Università di Milano, Milan, Italy; ^4^Geriatric Unit, Fondazione IRCCS Ca' Granda Ospedale Maggiore Policlinico, Milan, Italy

**Keywords:** aging, body composition, malnutrition, muscle aging, sarcopenia, diet, exercise, signaling pathways

Advancing age is characterized by remarkable changes in body composition that are not necessarily reflected by modifications in body weight (1). One notable body reshaping observed during aging is sarcopenia—the decline in muscle mass, function, and strength that increases the risk of incurring negative health-related outcomes (e.g., disability, loss of independence, institutionalization, death) (2). The loss of lean body mass is often masked by stable or even increased body weight as a consequence of greater adiposity, potentially resulting in the detrimental condition of sarcopenic obesity (3).

Sarcopenia is a “hot topic” in geriatrics. Nevertheless, to date, the condition is still orphan of a univocal operational definition (2). The matter is further complicated by the heterogeneity of the clinical phenotypes, the complex pathophysiology, and the lack of standardized biomarkers. As frequently occurring with geriatric syndromes, the frequent superimposition of common conditions of advanced age complicates the study of a sarcopenia as a stand-alone disease (4). The ultimate consequences of such an impasse are the lack of incorporation of sarcopenia in everyday clinical practice and the absence of pharmacological treatments against the condition.

The research collection “Age-Related Changes in Body Composition: Mechanisms, Clinical Implications, and Possible Treatments” aimed at gathering contributions on age-related changes in body composition from different, yet complementary, points of view by convening clinicians and basic researchers working in the field of muscle aging in human and preclinical models.

In an up-to-date overview, Ziaaldini et al. illustrated the putative molecular mechanisms underlying the onset and progression of sarcopenia. These include biochemical pathways pertaining to insulin-like growth factor 1 (IGF-1)/Akt/mammalian target of rapamycin (mTOR), forkhead box O (FoxO) transcription factors, transforming growth factor (TGF)-β, nuclear factor (NF)-κB, and the mitogen-activated protein kinases (MAPKs). The effects of exercise on some of these signaling pathways are also discussed (Ziaaldini et al.).

Sarcopenia was also framed in the context of neuromuscular aging, focusing on dysfunctional neuromuscular junctions (NMJs) as a mechanism involved in muscle wasting by Casati et al. In this context, the dissection of brain–muscle cross talk during aging may help unveil novel biomarkers for the condition (Casati et al.). The synaptosomal-associated protein of 25 kDa (SNAP25) was found to accumulate in the plasma membrane of nerve terminals at NMJs and regulate exocytosis of peripheral and central synapses in preclinical models (Casati et al.). Hence, SNAP25 may be a relevant pathway for understanding the molecular mechanisms regulating muscle homeostasis during aging, potentially supporting the clustering of biological determinants in sarcopenia.

Malnutrition, in particular, disarrangements in protein–amino acid metabolism, is a geriatric giant contributing to poor muscle function in older adults, especially in frail older patients (5, 6). As such, protein malnutrition is an actively investigated mechanism for the pathogenesis of sarcopenia. Nutritional interventions (as stand-alone interventions or in association with physical activity programs) are currently the most effective strategies for the management of sarcopenia (7). It is well-known that overall inadequate protein intake can impinge on muscle protein balance (i.e., affecting synthesis and degradation) and lead to skeletal muscle atrophy. However, only recently, it has become clear that protein quality matters as well. Indeed, the administration of diets with slightly imbalanced essential amino acids (EAA)/non-EAA blends to late middle-aged mice induced body weight loss and tissue wasting, especially muscle atrophy (Corsetti et al.). Thus, an adequate EAA/non-EAA ratio is likely necessary for preserving whole-body energy status (Corsetti et al.).

Finally, the thickness of the quadriceps muscle assessed by bedside ultrasound was shown to predict rehospitalization and functional decline in older adults (Guerreiro et al.). Hence, this measure was indicated as a valuable tool for the evaluation of bedridden hospitalized older adults. In the same study, a contractile index was elaborated and associated with functional decline 3 months after hospital discharge (Guerreiro et al.).

Thanks to its multidisciplinary deployment, this collection provides an opportunity to increase awareness on the relevance of age-related changes in body composition and expand our knowledge on the pathophysiology of muscle aging.

## Author Contributions

All authors listed have made a substantial, direct and intellectual contribution to the work, and approved it for publication.

## Conflict of Interest

The authors declare that the research was conducted in the absence of any commercial or financial relationships that could be construed as a potential conflict of interest.
